# Association between dry eye disease and asthma: a nationwide population-based study

**DOI:** 10.7717/peerj.5941

**Published:** 2018-12-05

**Authors:** Yung-Chieh Huang, Wei-Cheng Chan, Jiaan-Der Wang, Lin-Shien Fu, Yu-Tse Tsan

**Affiliations:** 1 Department of Pediatrics, Taichung Veterans General Hospital, Taichung, Taiwan; 2 Division of Pediatrics, Puli Branch, Taichung Veterans General Hospital, Nantou, Taiwan; 3 Department of Emergency Medicine, Taichung Veterans General Hospital, Taichung, Taiwan; 4 School of Medicine, China Medical University, Taichung, Taiwan; 5 Institute of Clinical Medicine, National Yang-Ming University, Taipei, Taiwan; 6 Department of Pediatrics, National Yang-Ming University, Taipei, Taiwan; 7 Institute of Occupational Medicine and Industrial Hygiene, National Taiwan University College of Public Health, Taipei, Taiwan; 8 School of Medicine, Chung Shan Medical University, Taichung, Taiwan

**Keywords:** Dry eye disease, Asthma, Taiwan, NHIRD, Allergy, Immunology, Allergic comorbidities

## Abstract

**Background:**

Dry eye disease (DED), a chronic ocular disease, is associated with numerous medical issues, including asthma. However, studies on these associations are limited. In this study, we investigated the incidence of DED among patients with asthma and its correlation with other allergic comorbidities.

**Methods:**

We retrospectively analyzed data from the National Health Insurance Research Database of Taiwan. We compared the data of 41,229 patients with asthma with those of 164,916 sex- and age-matched non-asthma controls. We followed up the patient and control groups from 1998 to 2010, and compared the rate of DED in these two groups. We further analyzed the allergic comorbidities and asthma-related medication use among the patients with asthma to verify whether these factors were associated with DED.

**Results:**

The patients in the asthma group were more likely to have DED than were the controls (6.35% vs. 4.92%, *p* < 0.0001). In the asthma group, female had a higher risk of DED (odds ratio (OR) = 1.70, 95% confidence interval (CI) [1.57–1.85]) than males did. After adjustment for sex, age, income, urbanization, and the other two allergic comorbidities, patients with allergic rhinitis (adjusted OR = 1.58, 95% CI [1.46–1.72]) and urticaria (adjusted OR = 1.25, 95% CI [1.12–1.38]) were more likely to have DED, but not patients with atopic dermatitis (adjusted OR = 1.17, 95% CI [0.98–1.40]). Patients with asthma who had prescriptions of leukotriene receptor antagonists (LTRAs) (adjusted OR = 1.29, 95% CI [1.01–1.64]), oral antihistamines (adjusted OR = 2.02, 95% CI [1.84–2.21]), and inhaled corticosteroids (adjusted OR = 1.19, 95% CI [1.04–1.36]) exhibited association with DED.

**Discussion:**

Our findings reveal that patients with asthma—particularly females—were more likely to have DED, with comorbidities such as allergic rhinitis and urticaria, and prescriptions including LTRAs, antihistamines, and inhaled corticosteroids. The results suggest that in clinical practice, physicians should pay attention to DED, particularly in patients with a high risk of DED.

## Introduction

Dry eye disease (DED) is a common and chronic ocular disorder. It is a multifactorial disease of the ocular surface and tear glands that results in long-term discomfort (Lemp et al., 2007; Lemp, 2008). DED is an immune- and inflammation-related disease (Stevenson, Chauhan & Dana, 2012; Wei & Asbell, 2014). DED is generally diagnosed according to its symptoms, without united criteria (Lemp et al., 2007). In several population-based studies, DED has been linked to numerous other medical issues including cardiovascular, neurological, rheumatological, endocrine, gastrointestinal, and mental diseases (Ahn et al., 2014; Galor et al., 2011; Uchino et al., 2011; Van Der Vaart et al., 2015; Vehof et al., 2014; Wang et al., 2012).

Asthma is also a common and chronic disease in adults and children, involving airway inflammation, obstruction, and hyperresponsiveness. Allergen-specific type 2 CD4^+^ T-helper cells and related cytokines mediate the inflammatory process, and several cells, including eosinophils, mast cells, macrophages, and epithelial cells, play essential roles in the pathogenesis of asthma (Locksley, 2010). Patients with asthma often have other allergic conditions including allergic rhinitis, allergic conjunctivitis, and atopic dermatitis (Ledford & Lockey, 2013; Neto et al., 2010). Several ophthalmologists have reported the association of DED with a high risk of allergy or asthma (Chia et al., 2003; Moss, Klein & Klein, 2008; Paulsen et al., 2014; Wang et al., 2012); however, the risk of DED in patients with asthma or allergy has not been investigated. The present study analyzed the possible factors associated with DED in asthma patients.

## Materials and Methods

### Database

The data used in this study were obtained from the National Health Insurance (NHI) Research Database of Taiwan. The NHI program, initiated in Taiwan in 1995, currently provides medical care to more than 98% of the total population of 23 million. Taiwan’s National Health Research Institute (NHRI) provides a representative database of 1 million randomly sampled patients from all NHI enrollees for research purposes. The identification information of every individual is encrypted before making the data publicly available for research purposes.

### Study sample

In this study, we retrospectively identified all patients with asthma from among the one million randomly sampled patients in the NHRI representative database between 1998 and 2010 by using the International Classification of Diseases, Ninth Revision, Clinical Modifications (ICD-9-CM) code 493. To ensure the validity of diagnoses, only those patients who received at least three asthma diagnoses as outpatients or one as inpatients were included in the analysis. Patients who were less than 18-years-old in 1997 were excluded from this study. The control group was randomly selected from the remainder of the NHRI representative database. Every patient with asthma had four sex-and age-matched controls according to birth year.

Dry eye disease was defined as three consecutive diagnoses of DED (ICD-9-CM code 375.15, tear film insufficiency, unspecified) and eye lubricant prescriptions. Patients with Sjogren syndrome (ICD-9-CM code 710.2) were excluded from the study.

### Allergic comorbidities and asthma-related medications

Allergic comorbidities, including allergic rhinitis (ICD-9-CM code 477), atopic dermatitis (ICD-9-CM codes 691.0 and 691.8), and acute urticaria (ICD-9-CM code 708), were defined as at least three diagnoses during the observation period. The prescription of asthma-related medications (leukotriene receptor antagonists (LTRAs), oral antihistamines, and inhaled corticosteroids) were defined as an outpatient prescription for more than 180 days during the observation period. The odds ratios (ORs) for allergic comorbidities and asthma-related medications were adjusted for sex, age, income, and residence area.

### Statistical analysis

All statistical analyses were performed using SAS (version 9.2; SAS Institute, Cary, NC, USA). We used the chi-squared test and *t*-test to compare the differences between asthma and the matched control groups. We assessed the risk factors for DED among patients with asthma by using ORs accompanying 95% confidence intervals (CIs); *p* < 0.05 were considered significant.

### Ethics

The design of this study was reviewed and approved by the Institutional Review Board of the Taichung Veteran General Hospital, Taiwan. (Approval number: IRB TCVGH No: CE16141B).

## Results

In total, data of 206,145 people were sampled in this study with 41,229 and 164,916 people comprising the asthma group and non-asthma groups, respectively. The distribution of sex and age for both groups is summarized in Table 1. During the observation period (1998–2010), 6.35% of patients received diagnosis of DED in the asthma group compared with 4.92% of patients in the non-asthma group (*p* < 0.0001). Patients with DED were younger in the asthma group than in the non-asthma group; the age distributions of patients with DED in the asthma and non-asthma groups were significantly different, as presented in Table 2.

**Table 1:** Characteristics of 41,229 patients with asthma and 164,916 non-asthma controls enrolled.

	Total (*n* = 206,145)		Asthma (*n* = 41,229)		Non-asthma (*n* = 164,916)		
	*n*	%	*n*	%	*n*	%	*p*-value
Male	94,430	45.81	18,886	45.81	75,544	45.81	1.000
Female	111,715	54.19	22,343	54.19	89,372	54.19	
Age in 1997 (years old)							
Mean (range)	49.56 (18–91.64)		49.56 (18–91.43)		49.56 (18–91.64)		0.967
STD	16.48		16.48		16.48		
IQR	36.33–63.22		36.34–63.22		36.32–63.22		

**Table 2:** Diagnosis of DED and age distribution of asthma and non-asthma groups.

	Total (*n* = 206,145)		Asthma (*n* = 41,229)		Non-Asthma (*n* = 164,916)		
	*n*	%	*n*	%	*n*	%	*p*-value
DED	10,726	5.2	2,619	6.35	8,107	4.92	<0.0001
Non-DED	195,419	94.8	38,610	93.65	156,809	95.08	
Age distribution of DED							
18 ≤ age < 40	489	4.56	157	5.99	332	4.1	<0.0001
40 ≤ age < 65	3,877	36.15	1,052	40.17	2,825	34.85	
Age ≥ 65	6,360	59.3	1,410	53.84	4,950	61.06	
Age of DED (years old)							
Mean	65.82		64.12		66.37		<0.0001
95%CI	65.58–66.06		63.61–64.62		66.09–66.64		
IQR	58.2–75.2		56.1–73.9		59.1–75.5		

We further analyzed the distribution and risk factors for DED in the asthma group (shown in Table 3). In the asthma group, female patients were more likely to develop DED than were male patients (OR = 1.70, 95% CI [1.57–1.85]). After adjustment for sex, age, income, urbanization, and allergic comorbidities, we found that patients with allergic rhinitis (adjusted OR = 1.58, 95% CI [1.46–1.72]) and urticaria (adjusted OR = 1.25, 95% CI [1.12–1.38]) were associated with DED, but not patients with atopic dermatitis (adjusted OR = 1.17, 95% CI [0.98–1.4]). Patients with asthma and DED had more prescriptions for LTRAs (adjusted OR = 1.29, 95% CI [1.01–1.64]), oral antihistamines (adjusted OR = 2.02, 95% CI [1.84–2.21]), and inhaled corticosteroids (adjusted OR = 1.19, 95% CI [1.04–1.36]) for more than 180 days.

**Table 3:** Sex distribution, allergic comorbidities, and medication used among DED and non-DED individuals in the asthma group.

		DED		Non-DED		Odds ratio		
		*n*	%	*n*	%	Value	95% CI	*p*-value
Sex								
	Male	888	33.91	17,998	46.61	Ref.		
	Female	1,731	66.09	20,612	53.39	1.70	1.57–1.85	<0.0001
Comorbidities								
	Allergic rhinitis	1,283	48.99	15,838	41.02	1.38	1.28–1.50	<0.0001
	Adjust*					1.58	1.46–1.72	<0.0001
	Atopic dermatitis	148	5.65	1,656	4.29	1.34	1.13–1.60	0.001
	Adjust*					1.17	0.98–1.40	0.081
	Urticaria	505	19.28	5,932	15.36	1.32	1.19–1.46	<0.0001
	Adjust*					1.25	1.12–1.38	<0.0001
Drug used more than 180 days								
	LTRAs	76	2.9	938	2.43	1.20	0.95–1.52	0.1312
	Adjust^#^					1.29	1.01–1.64	0.0401
	Oral antihistamine	1,940	74.07	22,019	57.03	2.15	1.97–2.36	<0.0001
	Adjust^#^					2.02	1.84–2.21	<0.0001
	Inhaled steroids	270	10.31	3,389	8.78	1.20	1.05–1.36	0.0077
	Adjust^#^					1.20	1.04–1.36	0.0109

**Notes:**

* Adjusted with sex, income, urbanization, age, and the other two allergic comorbidities.

# Adjusted with sex, income, urbanization, and age.

Table 4 presents the distribution and risk factors for DED in the non-asthma group. Female patients were more likely to develop DED than were male patients (OR = 1.77, 95% CI [1.69–1.86]). We found that allergic rhinitis, urticaria, and atopic dermatitis were associated with DED after adjustment for sex, age, income, urbanization, and allergic comorbidities.

**Table 4:** Sex distribution and allergic comorbidities among DED and non-DED individuals in the non-asthma group.

		DED		Non-DED		Odds ratio		
		*n*	%	*n*	%	Value	95% CI	*p*-value
Sex								
	Male	2,666	32.89	72,878	46.48	Ref.		
	Female	5,441	67.11	83,931	53.52	1.77	1.69–1.86	<0.0001
Comorbidities								
	Allergic rhinitis	1,704	21.02	17,330	11.05	2.14	2.03–2.27	<0.0001
	Adjust*					2.16	2.04–2.29	<0.0001
	Atopic dermatitis	284	3.50	3,888	2.48	1.43	1.26–1.61	<0.0001
	Adjust*					1.17	1.03–1.33	0.0144
	Urticaria	1,052	12.98	14,924	9.52	1.42	1.33–1.52	<0.0001
	Adjust*					1.24	1.16–1.33	<0.0001

**Note:**

* Adjusted with sex, income, urbanization, age, and the other two allergic comorbidities.

## Discussion

In this study, we found that patients in the asthma group were more likely to have DED than were those in the control group. Several allergic comorbidities and medications were associated with DED among both asthma and non-asthma patients; these findings have rarely, if ever, been discussed in previous studies.

The prevalence of DED varies from 5.5% to 33.7% (Gayton, 2009; Han et al., 2011; Um et al., 2014) between different populations, primarily affecting women, and elderly people (Lin et al., 2003; McCarty et al., 1998; Moss, Klein & Klein, 2000). The mechanisms underlying the relationships between DED and several reportedly associated medical conditions remain unclear. The percentage of DED in our study is similar to that in other studies.

In the Blue Mountains Eye Study conducted by Chia et al. (2003), patients with more than one of the four DED symptoms were more likely (OR = 1.6) to have asthma (dry eye, grittiness, itchiness, and discomfort). In a nationwide population-based study, Wang et al. (2012) found that patients with DED are associated with asthma (6.18% vs. 5.01%, *p* < 0.001). In a 10-year cohort study, Moss, Klein & Klein (2008) found that elderly patients with an allergy history had an increased incidence of DED (25.2% vs. 20.4%, *p* = 0.006). In this study, we found a similar association between asthma and DED.

In the present study, we found an even higher association of DED in patients with asthma with other allergic comorbidities; this finding has not been reported before although the association of single allergic diseases with DED has been reported in a few studies. Dogru et al. (2016) found that children with allergic diseases had a shorter tear film break-up time than did healthy children, which may lead to DED in the future. A short tear film break-up time leads to the disruption of the protective barrier against possible airborne pollutants or allergens, which may further induce or aggravate allergic conjunctivitis. Yenigun et al. (2016) have described a significant association between DED and allergic rhinitis using a positive skin-prick test. Apart from these studies, the relationship between allergic symptoms and DED has rarely been discussed. Atopic dermatitis was once believed to be the first presentation of the classical “atopic march.” However, nowadays, abnormalities of the epidermal structure and function are considered crucial pathophysiological peculiarities of atopic dermatitis rather than only secondary effects of immunological mechanisms (Weidinger & Novak, 2016). This observation may explain our findings that atopic dermatitis is associated with DED in the asthma group, but the association is not as prominent after adjustment for the other two comorbidities.

In the Beaver Dam Offspring Study conducted by Paulsen et al. (2014), a history of allergies and the use of medications, including antihistamines and steroids, were found to be risk factors of DED. However, the authors did not further discuss the mechanisms underlying the association between DED and those medications. In this study, we specifically analyzed the medication associated with asthma, which has rarely been discussed in the literature. LTRAs and inhaled corticosteroids are common asthma controllers. We found that patients with asthma with prescriptions for these three types of medication for more than 180 days were associated with DED, after adjustment for sex, age, income, and urbanization. Due to the limitation of this study, determining whether these medications increased the risk of DED or whether patients with more severe asthma symptoms consumed more medication and had more frequent DED was difficult. Oral antihistamines are not regularly used in treatment of asthma, but they are widely prescribed for other allergic symptoms. The relationship between antihistamines and DED has been described in several studies (Galor et al., 2011; Moss, Klein & Klein, 2008; Paulsen et al., 2014), possibly because of the mucosa-drying effect of the antihistamines. In this study, we found a similar association between antihistamines and DED.

Although the exact mechanisms underlying both DED and asthma are not completely clear, we believe that some correlations may exist between the pathogenesis of the two diseases. Inflammation is now hypothesized to be the core mechanism underlying DED. Inflammatory cells, cytokines, and chemokines were detected on the ocular surface and in tears of patients with DED (Wei & Asbell, 2014). By contrast, asthma is triggered by exposure to allergens, infection, or irritants. Innate and adaptive cells, including eosinophils, mast cells, lymphocytes, neutrophils, and monocytes, are activated to produce cytokines and chemokines and enhance inflammation and airway hyperresponsiveness.

This study has limitations. This is a retrospective study using the database provided by NHRI; therefore, the patients’ personal history, environmental exposure, and severity of asthma or DED were not available in the database. We used the ICD-9-CM diagnoses codes and medication prescription to define DED, asthma, and allergic comorbidities. For validation, we defined DED as three consensus diagnoses of DED and eye lubricant prescription to confirm the diagnosis (Wang et al., 2012). We defined medication usage as prescription for more than 180 days during the study period; however, the amount of each type of medication was not calculated as a defined daily dose in our study. Several environmental factors, such as smoking and exposure to certain pollutants, can be important risk factors for both asthma and DED, but they cannot be analyzed in the study.

## Conclusions

In conclusion, our study reveals that patients with asthma were more prone to DED than were the matched controls. In the asthma group, female patients, as well as patients with allergic comorbidities, including allergic rhinitis, atopic dermatitis, and urticaria, were more likely to have DED than were those without comorbidities. The use of oral antihistamines, LTRAs and inhaled corticosteroids was associated with DED. Asthma is a chronic disease that requires long-term follow-up; the results of our study suggest that in clinical practice, physicians should pay attention to DED particularly in patients with a high risk of DED.

## Additional Information and Declarations

### Data Availability

The following information was supplied regarding data availability: Raw data for this work was obtained by application from the National Health Insurance Research Database, Taiwan (http://nhird.nhri.org.tw/en/index.html) and may not be shared according to the Database’s rules governing use. Access to the data used in this study may be obtained by citizens of Taiwan who fulfill the requirements of conducting research projects.
